# Emergent coordination in temporal partitioning congestion games

**DOI:** 10.1371/journal.pone.0308341

**Published:** 2024-08-19

**Authors:** Reuven Cohen, Oren Perez

**Affiliations:** 1 Department of Mathematics at Bar-Ilan University, Ramat Gan, Israel; 2 Faculty of Law, Head, Bar-Ilan University Multidisciplinary School for Environment and Sustainability, Bar-Ilan University, Ramat Gan, Israel; LUMSA: Libera Universita Maria Santissima Assunta, ITALY

## Abstract

In this article we study the social dynamic of temporal partitioning congestion games (TPGs), in which participants must coordinate an optimal time-partitioning for using a limited resource. The challenge in TPGs lies in determining whether users can optimally self-organize their usage patterns. Reaching an optimal solution may be undermined, however, by a collectively destructive meta-reasoning pattern, trapping users in a socially vicious oscillatory behavior. TPGs constitute a dilemma for both human and animal communities. We developed a model capturing the dynamics of these games and ran simulations to assess its behavior, based on a 2×2 framework that distinguishes between the players’ knowledge of other players’ choices and whether they use a learning mechanism. We found that the only way in which an oscillatory dynamic can be thwarted is by adding learning, which leads to weak convergence in the no-information condition and to strong convergence in the with-information condition. We corroborated the validity of our model using real data from a study of bats’ behaviour in an environment of water scarcity. We conclude by examining the merits of a complexity-based, agent-based modelling approach over a game-theoretic one, contending that it offers superior insights into the temporal dynamics of TPGs. We also briefly discuss the policy implications of our findings.

## Introduction

Congestion, a situation in which the demand for a resource exceeds its capacity, constitutes a pressing global problem. As the world population continues to grow, congestion dilemmas become more prevalent both in the social world (e.g., traffic jams, crowded natural reserves) and in the natural world (species competing for dwindling resources because of human interference and climate change). Congestion poses a significant governance and regulatory challenge [1: 28, 2:93]. Temporal partitioning games (‘**TPGs**’), are a special class of congestion dilemmas in which participants must coordinate an optimal time partitioning for using a limited resource with limited external intervention. Whether TPGs can converge to optimal or close-to-optimal equilibrium without external intervention is a challenging theoretical problem with important policy implications.

In this paper, we analysed the social dynamic of TPGs. We use the analysis of TPGs to compare a complexity-based approach with a game-theoretic, analytic modeling approach to social dilemmas [3, 4]. We begin by describing the structure of TPGs, explain why they create a social dilemma, and highlight their potential welfare-destructive dynamic. Despite the prevalence of TPGs, their unique dynamic received relatively little attention in the economic and ecological literature. We then develop a model that captures the dynamic of these games, focusing on what we call the ‘Black Iris Blossom Game’. We ran simulations to test the model behaviour in a 2×2 framework that distinguishes between the players’ access to information and the use and non-use of learning. We further test our model based on real data from a study of bats’ behaviour. We demonstrate that adopting a complexity-based approach, using agent-based modelling, provides better insights into the temporal dynamic of TPGs than the game-theoretic approach.

In congestion games, agents must coordinate how to jointly use a limited resource most beneficially [5: 147]. A subclass of congestion games, which is the focus of this article, concerns games in which users can partition their use patterns over time to achieve optimal joint usage of a resource (users may also optimize joint usage via spatial partitioning, which we do not explore in this study). A unique feature of congestion games is the presence of externalities: a situation in which the action of each agent adversely affects the utility of the other agents using the resource. This adverse effect can be either purely *pecuniary*, where an increase in the number of users reduces the benefit received by each user because of the limited capacity of the resource, or *real*, where the congestion leads to the physical destruction of the resource [5: 147–148]. A further unique feature of temporal partitioning games is that they can give rise to a potentially destructive cyclical reasoning pattern that could undermine the capacity of the group to achieve optimal equilibrium. This aspect of congestion games was first pointed out by Brian Arthur in his El Farol bar model [4, 6].

Below we discuss three examples of temporal partitioning games that illustrate their potentially destructive meta-reasoning pattern. These games occur in both the social and natural world; their analysis should therefore be of interest to biologists, ecologists, and social scientists alike. One example is natural recreation goods in which people compete for visiting opportunities in a resource that can only host a limited number of visitors [7]. The dilemma of nature lovers is how to choose a visiting time (day, hour) to maximize their enjoyment. We assume that people’s ‘nature experience’ decreases as the visiting site becomes more crowded, up to a certain threshold level that is a function of spatial density. If the number of visitors exceeds that level, people receive no utility from the visit, and may even experience negative utility if we take into account their disappointment and travelling costs [8, 9]. The erosion in the quality of the recreation experience, which takes place as the number of visitors increases, represents a pecuniary externality because the natural asset itself is left unharmed. TPGs may also involve a real externality when overcrowding not only reduces the visitors’ utility but also causes harm to the natural good (the harm may be temporary, if the ecological asset replenishes itself, or permanent) [7: 419–20]. The real externality can be captured by a ‘resilience’ threshold that represents the capacity of the habitat to withstand stress.

TPGs can also involve purely social goods. A good example is competition for scarce publication slots. A case in point is the U.S. legal academic market. In contrast to most scientific journals, which accept submissions year around, U.S. law reviews receive articles for publication in two cycles that take place annually between mid-February to mid-March and mid-August to mid-September [10, 11]. The unique temporal feature of the U.S. law reviews market imposes an enormous burden on the editors of the journals. Each year, U.S. law reviews receive thousands of submissions that compete for limited slots, especially in top-tier journals [12: 636, 13: 193]. The submission game involves two scarce goods that jointly create a congestion dynamic: a limited number of slots in which articles can be published and the editors’ limited attention capacity. Authors face the dilemma of selecting an optimal submission date for their article to maximize the probability of its acceptance, considering the two constraints. The exact dynamics of the submission game depend on various other variables, but these contextual factors do not alter its fundamental congestion structure.

The dilemma underlying the above examples is whether people can self-organize to optimally partition their usage patterns (visiting times or submission times), which will maximize the total utility of the group. Reaching an optimal solution could be undermined, however, by a collectively destructive, self-nullifying, meta-reasoning pattern [14]. Let us assume that everyone’s priority is to visit the natural reserve or submit the paper on the least crowded day. Assume further that there is a focal day that is preferred by everyone because of some collective (folk) belief that it is in some way the ‘best’ day (e.g., the day on which the flowers at the reserve are at their peak blossom or editors are supposed to be particularly receptive). But because this is what everyone prefers, this focal day is likely to be over-crowded, providing negative utility to anyone who attempts to use the resource at that time. As people may anticipate that the focal day is likely to be the first choice of everyone, they may prefer to choose another day, for example, the day before or after the focal day. But if everyone follows this reasoning pattern, these two days will also become overcrowded. Below we explore whether a collectively beneficial temporal partitioning equilibrium can emerge under these conditions despite this potentially destructive meta-reasoning pattern.

TPGs can also occur in the natural world. Consider how biologically similar (same taxonomic class, similar body mass) and ecologically similar species (those utilizing the same resource) use water resources in environments in which water is scarce [15]. The question is whether such species can coordinate their visiting times to water resources that are too small to accommodate simultaneous use by spatial separation, achieving optimal temporal partitioning. The dynamic of TPGs in the animal world differs from social TPGs in two aspects: (a) because animals do not possess the human capacity for strategic reflection, the destructive cyclical dynamic that is a problematic feature of human interaction in TPGs is probably not an issue; and (b) unlike humans, animals have no legal mechanisms that can be invoked to resolve coordination problems. Despite the differences, on situations in which there is limited regulatory intervention we believe that the dynamics of social and animal TPGs could be very similar [16].

## Model and methods

To reveal the dynamic of TPGs, we developed a model that focuses on the temporal usage of a limited natural recreation good. Although our model is based on a particular socio-ecological dilemma, it can be applied to any TPG with multiple interacting agents and limited regulatory oversight. It can thus be considered as a prototype model for any TPG. At the core of our model, is the *Iris atropurpurea* (the ‘black iris’), an endangered species that can be found in a few small enclaves, with a total area of less than 1 km^2^, across the coastal area of Israel [17: 158].

This geographic dispersion makes the black iris particularly vulnerable to urban development pressures [17, 18]. The exceptionally eye-catching flowers of the black iris attract many visitors during the time of its blossoming. One of the most attractive habitats of the black iris is situated in the calcareous hills of Ness Ziona, a small town in the centre of Israel. The flowering season of the black iris stretches from late January to the end of February and reaches its peak around mid-February [18: 398, 19: 972]. The short blossom season together with the limited areas in which the black iris can be found creates a congestion problem, which we call the *‘Black Iris Blossom Game’*. The dilemma for iris lovers is how to choose a day to maximize their enjoyment of the blossom. We assume that people’s ‘nature experience’ decreases as the visiting site becomes more crowded, up to a certain threshold level of density. If the number of visitors exceeds that level, people receive no utility from the visit and may even experience negative utility if we take into account their disappointment and travelling costs [8, 9]. As the number of visitors increases, the erosion in the quality of the recreation experience represents a pecuniary externality because the natural asset itself is left unharmed. We assume that all things being equal, people prefer to visit the iris habitat when the ratio of people/flowers is minimal. The blossom game may also involve real externality that can occur when overcrowding not only reduces the visitors’ utility but also causes harm to the flowers themselves [7: 419–20] (The harm could be seasonal if the iris population recovers in the next season, or it could be permanent). We focus in our model only on the pecuniary externality. We abstracted away real-life constraints, such as the distinction between weekdays and weekends, for the analysis.

The dilemma underlying this hypothetical scenario is whether people can self-organize to optimally partition their visiting times so that the total enjoyment level of the group is maximized. As we argued above, reaching an optimal solution may be undermined, however, by a collectively destructive meta-reasoning pattern, which could lead to an endless oscillatory dynamic.

We develop our model and simulation in three steps:

1. We start with a base case in which players have no information about the visiting patterns (both historical and real time) and exercise no learning, to establish the foundational dynamics of the game. The base case also includes heterogeneity in the players’ preferences (represented by the utility they experience from visiting the nature reserve).
2. Next, we examine how the provision of information regarding the temporal visiting distribution in the previous season, affects the dynamics of the game and the prospects of convergence.
3. Finally, we introduce learning into both scenarios (base case and information disclosure) and examine how it affects the dynamics of the game and the prospects of convergence.

As elaborated below, we do not attempt to directly model the strategic aspect of the agents’ reasoning process (that is, their beliefs about the beliefs of other players). We address this issue by incorporating randomness into the players’ strategies. We return to this point in the discussion, contrasting our approach with the K-level reasoning model. Our model differs from Brian Arthur’s El Farol model in that agents do not form explicit expectations about what will be the attendance in the next blossom period but decide based on the success of their strategy in the previous period, and when available, about their potential success in the next period, given the distribution of visitors in the previous period when such data are available. This type of learning can be described as stimulus-response learning or reinforcement learning, which is about understanding how agents might learn to make optimal decisions through repeated experiences [20–22].

The game unfolds according to the following rules. After the first round, each player, *i*, collects a revenue, calculated using the formula:

$$R=f\left(d_{i},n\left(d_{i}\right),m_{i},C_{i}\right)$$ (1)

where *d*_*i*_ is the day chosen by player *i*, *n*(*d*_*i*_) is the number of players that chose the same day (i.e., *d*_*i*_), and *m*_*i*_ is a parameter of player *i* indicating the player’s preference (the degree to which the player values the experience of visiting nature). In the simulation, we tested only the *heterogeneous case*, where players have different preferences (some may appreciate nature more than others). *C*_*i*_ represents the disappointment cost of player *i* from having to return without seeing the flowers if the threshold is exceeded (for the sake of simplicity, we disregard the travel costs to the nature reserve), that is, the negative emotion experienced when the chosen option (to visit nature rather than stay at home) fails [23, 24]. *f* decreases as n(d_i_) increases and becomes negative for a large *n*(*d*_*i*_), depending on an exogenous variable, *z* that represents the capacity of the natural asset (the iris flowers habitat) to generate recreational utility for visitors. We assume that if the number of visitors reaches a certain threshold that reflects the visitors’ varying preferences for nature, the utility of that agent becomes negative: *f* = −*C*_*i*_ (in contrast to other common-pool choice models, we assume then that the pool is not infinitely divisible). *f* is also dependent on the strength of the players’ recreational preferences. This means that people who visit the flowers may receive different utility levels. *z* changes with the number of flowers that are available each day during the blossom period. *K* represents the distribution of flowers (*K*(*d*) on day *d*. The following rules describe the utility that players receive as a function of the number of flowers and the number of other attending visitors.


$$m_{i}\geq 1\;for\;each\;i\;(\text{a}\;\text{larger}\;m_{i}\;\text{represents}\;\text{stronger}\;\text{preference}\;\text{for}\;\text{nature})$$ (2)

$$\begin{array}{l} f\left(d_{i},n\left(d_{i}\right),m_{i},C_{i},\;Ki\right)=\;m_{i}\frac{K\left(d_{i}\right)}{n\left(d_{i}\right)}\;\text{if}\;m_{i}\frac{K\left(d_{i}\right)}{n\left(d_{i}\right)}\geq z*(f,\text{the}\;\text{utility}\;\text{each}\;\text{agent}\;\text{receives}\\ \text{from}\;\text{visiting}\;\text{the}\;\text{flowers},\;\text{increases}\;\text{with}\;m_{i}\;\text{and}\;\text{decreases}\;\text{with}\;n\left(d_{i}\right)). \end{array}$$ (3)

$$(1)\;f\left(d_{i},n\left(d_{i}\right),m_{i},C_{i},\;Ki\right)=0\;\;\left(\text{players}\;\text{who}\;\text{stay}\;\text{home}\;\text{receive}\;\text{zero}\;\text{utility}\right)$$ (4)

$$(2)\;f\left(d_{i},n\left(d_{i}\right),m_{i},C_{i},\;Ki\right)=-C_{i}\;\text{if}\;m_{i}\frac{K\left(d_{i}\right)}{n\left(d_{i}\right)}<z$$ (5)

The simulation included *n* agents (individuals), each with a preference attribute *m*_*i*_, chosen uniformly in the range [1,3]. It was based on a 2×2 framework that distinguishes between the players’ access to information and whether they use learning.

### Condition 1: Players have access only to local knowledge

#### Information set

Players have access only to data regarding the number of people who were present on the day they visited the habitat.

#### Strategy

Each player starts by randomly choosing a day from the blossom period, assigning a different weight to each day, proportional to the distribution of flowers over the blossom season. After each season, players assess their utility. If the utility is positive, they continue with the *same choice* the next season. If the utility is *zero* or *negative*, they choose a behaviour for next season based on the following rule:

With probability 1 − *p*, stay at home. With probability *p*, choose a random day (not including the home option) for the next season with some *weighted* (blossom dependent) probability.

In the second step of the ‘no-information’ condition, we added a learning mechanism with the following structure: if a player experiences negative utility, it multiplies *p* by a constant *L<1* (L was set in the simulation to 0.5) (the value of L affects the convergence rate but has little impact on the final result; if a player stayed home for several seasons, p would only be multiplied by L once). The learning mechanism means that players become increasingly less inclined to explore other visiting days after repeatedly experiencing negative results.

### Condition 2: Players have access to global knowledge (information disclosure by central planner)

#### Information set

Each player knows the total number and distribution of players who attended the habitat in the previous period (they do not have real-time information).

#### Strategy

Each player starts by randomly choosing a day from the blossom period, assigning a different weight to each day, proportional to the distribution of flowers over the blossom season. After each season, players assess their utility. If the utility is positive, they continue with the *same choice* the next season. If the utility is *zero* or *negative*, they choose a behaviour for next season based on the following rule:

With probability 1 − *p*, stay at home. With probability *p*, choose the option that produces the highest expected utility by calculating its expected utility for each day, based on adding itself to the last known attendance (the best option could be staying at home if all days are expected to produce negative utility).

The full-information scenario assumes that players have stronger cognitive capacities: they can evaluate a large information set and perform optimizing calculations. In the second step of the ‘with-information’ condition, we added a learning mechanism with the same structure of the one described above.

### Analytical analysis of the blossom game: Nash equilibrium

We distinguish first between optimal and sub-optimal Nash equilibria (NE). The class of optimal NE takes the following pattern: the blossom days are first filled by the players in the order of their preference strength (if some players have identical preferences, they will be chosen randomly), until no player can be added without incurring negative utility. All NE that satisfy these conditions represent social optimum. The second class of sub-optimal NE consists of situations in which the days are randomly populated by players, and although some agents with higher preferences stay at home, the allocation constitutes an NE. For instance, in an *n* player game, an NE may have *m* players (*m* < *n*) with a lower preference visiting the flowers if there is a player with a higher preference who chooses not to visit. In such a case, the NE is secured because the preference of the player who stays at home is not high enough to make his utility positive if he joins on any of the days (its expected revenue, *R*, for joining each of the blossom days, i.e., for *n*(*d*_*i*_) + 1 visitors, is negative given his preference profile, whereas the revenue of each of the other players is positive for their preference profiles for *n*(*d*_*i*_) visitors, therefore none of the players have an incentive to change their choices(. There could be multiple suboptimal NEs.

A drawback of NEs, both optimal and sub-optimal, is that they lack fairness. There is a small group of players (in the optimal NE class it consists of those with the highest preference profile) who receive a positive revenue every season, while the revenue for the rest of the players is always zero. This is a winner-takes-all solution, where a possibly large portion of society is prevented from accessing the common resource because of the stickiness of the NE solution. A key difficulty of the analytical approach is that it does not provide any insight into the social dynamics by which the system can converge (or fail to converge) to one of the multiple equilibria identified by the analysis. The computational approach allows us to shed light on the dynamics of temporal partitioning games, going beyond the limits of the analytical approach.

## Results

We used the following parameters for the simulation:

*m*_*i*_ = 1 + *U*, where *U* is chosen uniformly in the range [0,2].

*C*_*i*_ = -1 for all players (the behaviour is independent of the specific value, as long as it is negative).

*z* = 0.1 for all players.

The length of the blossom period is 19 days, with the blossom for each day being *K*(*d*_*i*_) = min{*i*, 20 − *i*}, i.e., a symmetric distribution achieving maximum on the mid-season day (k_10_ = 10).

The initial probability (*p*) of exploring alternative visitation slots was set as *p* = 0.5 for all the scenarios.

In all cases, we assume satisficing, i.e., all the players experiencing positive utility *do not* attempt to change their choice, even if a higher utility option is apparent.

### No-information condition without learning

**Fig 1:**
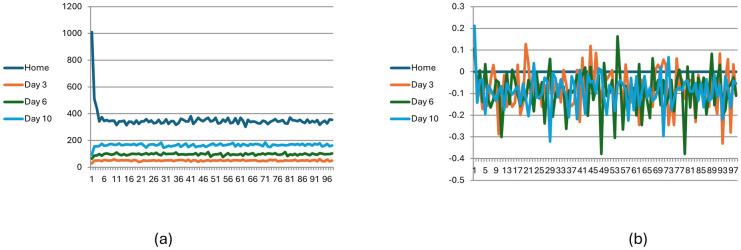
Visitation pattern in no-information, no-learning condition (N = 2000, p = 0.5). Fig 1(a) describes the number of visitors in selected days for 2,000 participants across 100 seasons with no learning (L = 1). Fig 1(b) describes the average revenue for the visitors for each day.

Fig 1(a) describes the number of visitors in selected days for 2,000 participants across 100 seasons with no learning (L = 1). Fig 1(b) describes the average revenue for the visitors for each day. As shown, equilibrium is not reached and there is over-crowdedness across the entire visitation period, with visitors suffering a negative utility each day. Generally, when the number of players is larger than the capacity of the iris habitat, the game follows an oscillatory dynamic and does not converge.

### No-information condition with learning

**Fig 2:**
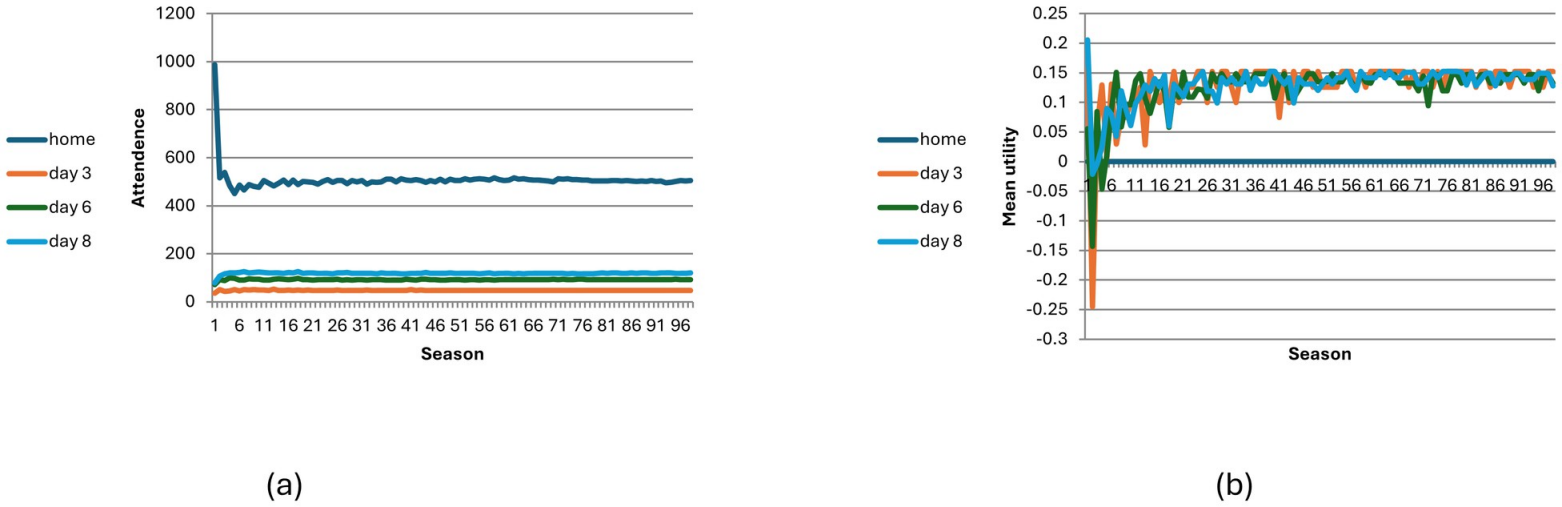
Visitation dynamic in the no-information with learning scenario across 100 seasons (N = 2000, p = 0.5). Fig 2(a) shows number of visitors in selected days; Fig 3(b) shows average utility per day.

Fig 2 shows how the game evolves in the no-information condition with 2,000 participants through 100 seasons with learning (*L* = 0.5). Number of visitors in selected days (Fig 2(a)) and average utility per day (Fig 2(b)). Adding learning leads to weak convergence, that is, the probability that players will change their behaviour between seasons becomes lower and lower and converges to zero. Mathematically, the probability of change between consecutive seasons approaches zero, but the probability that from a certain season onward no changes will occur is also zero. Although the composition of the people who stay at home every season varies, the variation rates become lower over time (see Fig 7 below and p. 2 in the supplementary materials).

### Full-information condition without learning

**Fig 3:**
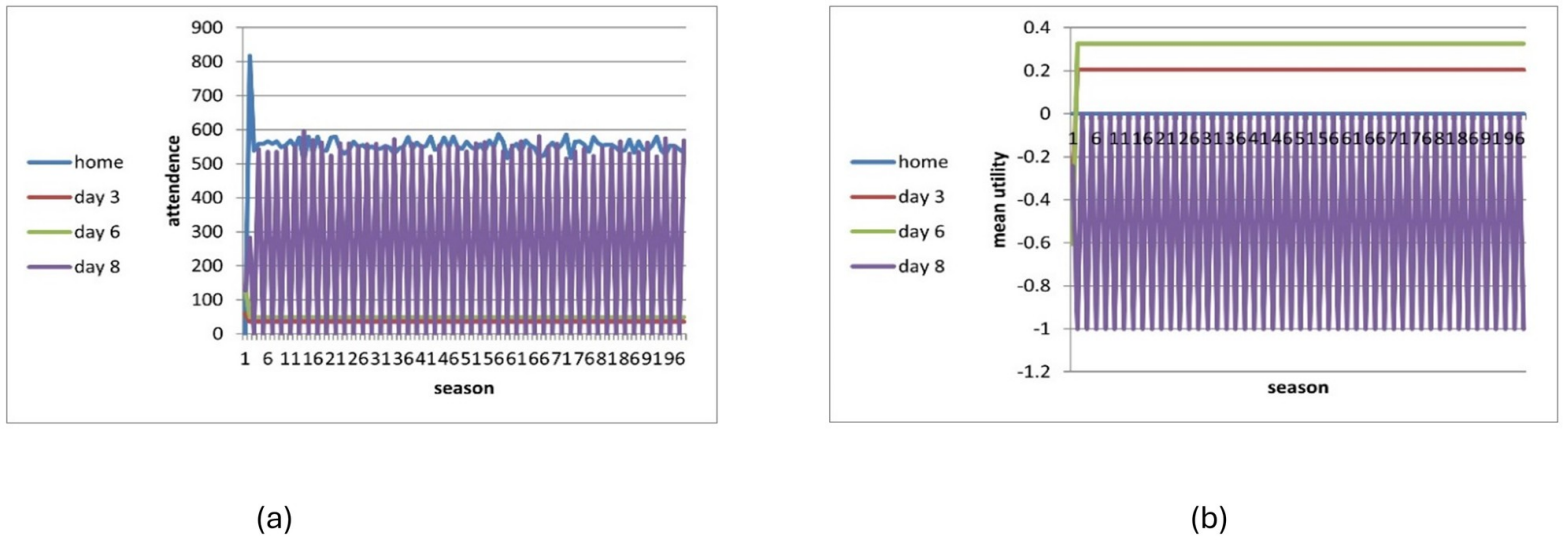
Visitation dynamic in the full-information no-learning condition (L = 1) across 100 seasons (N = 2000). Fig 3(a) shows the number of visitors in selected days; Fig 3(b) the average utility per day.

Fig 3 shows plots for the number of visitors in selected days (Fig 3(a)) and the average utility per day (Fig 3(b)) in the full-information and no-learning model (*L* = 1). The average utility remains negative, and the results keep fluctuating.

**Fig 4:**
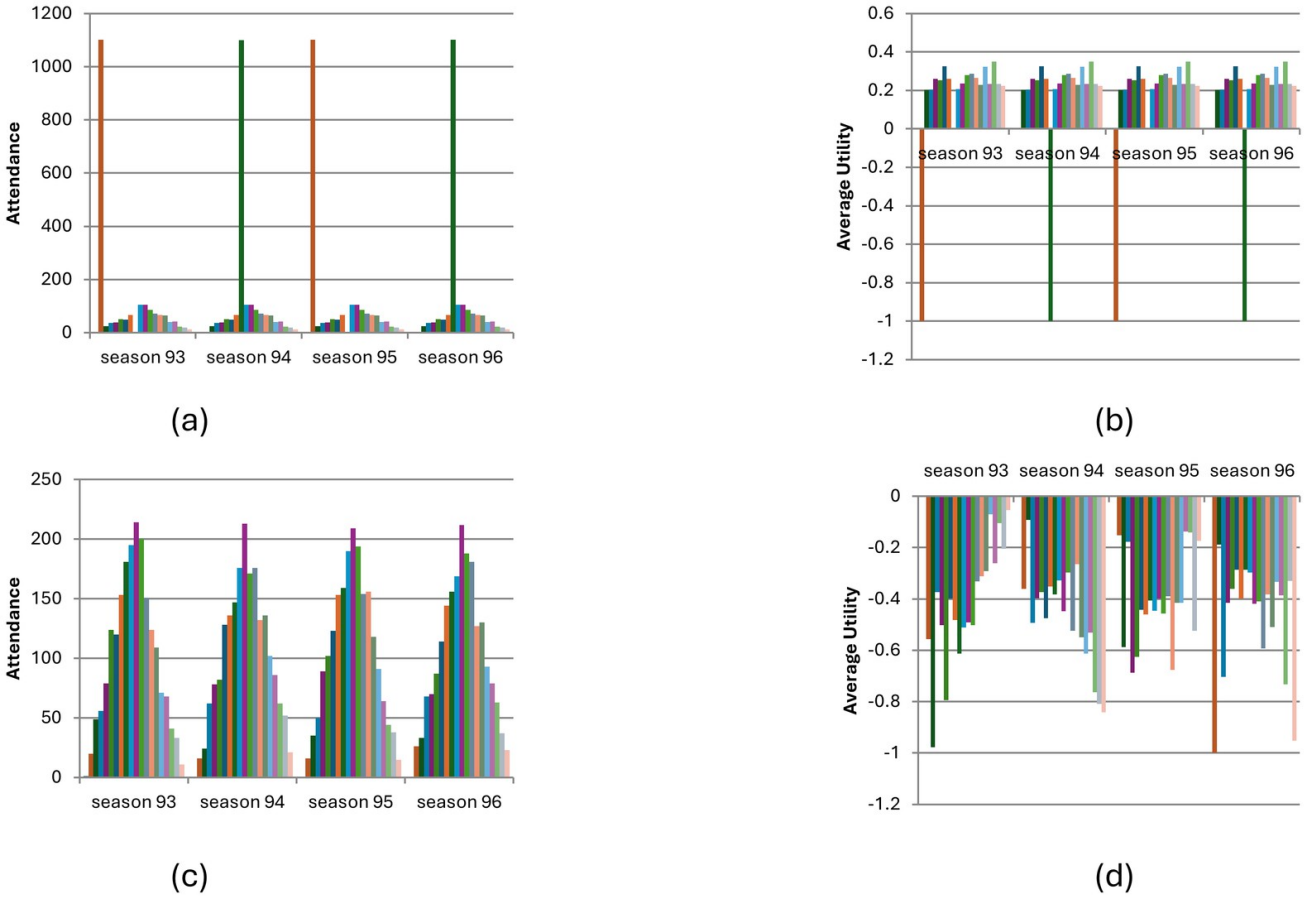
Visitation dynamic in the no-information (c,d) and full-information (a,b) no-learning conditions across 4 consecutive seasons (all days) (N = 2000).

Fig 4a and 4b shows the number of visitors with 2000 players and average utility per day with no learning (*L* = 1) with information. Four consecutive seasons are shown. The oscillations continue forever.

The no-information and full-information conditions differ in their oscillation patterns. In the no-information model (Fig 4c and 4d) players oscillate randomly between days but the number of visitors in each day remains approximately constant between seasons. This is due to the law of large numbers, stating that if the number of players is large enough, the number of players per day will be, with high probability, close to the expected value. However, the numbers do fluctuate, and especially in days with low attendance these fluctuations may be significant percentage-wise. In the full-information model, after a day becomes congested, all the players move to a non-congested day (including home) and continue oscillating without being able to break this pattern (Fig 4a and 4b). The results are shown for seasons 93–96. However, the oscillating behaviour starts at an early stage, after 3 or 4 seasons, and remains approximately stable.

### Full-information condition with learning

**Fig 5:**
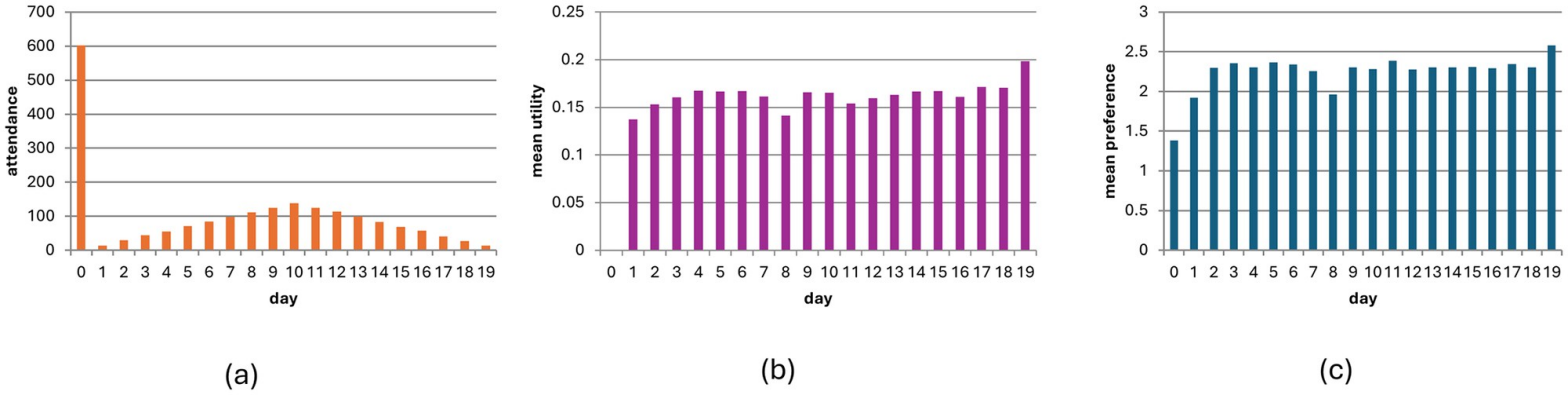
(a,b). Visitation pattern in the full-information with learning condition (N = 2000, after 100 seasons). Fig 5(a) distribution of visitors for each day; Fig 5(b) average utility; Fig 5(c), average preference of visitors per day (full-information, with learning condition) (N = 2000, after 100 seasons).

**Fig 6:**
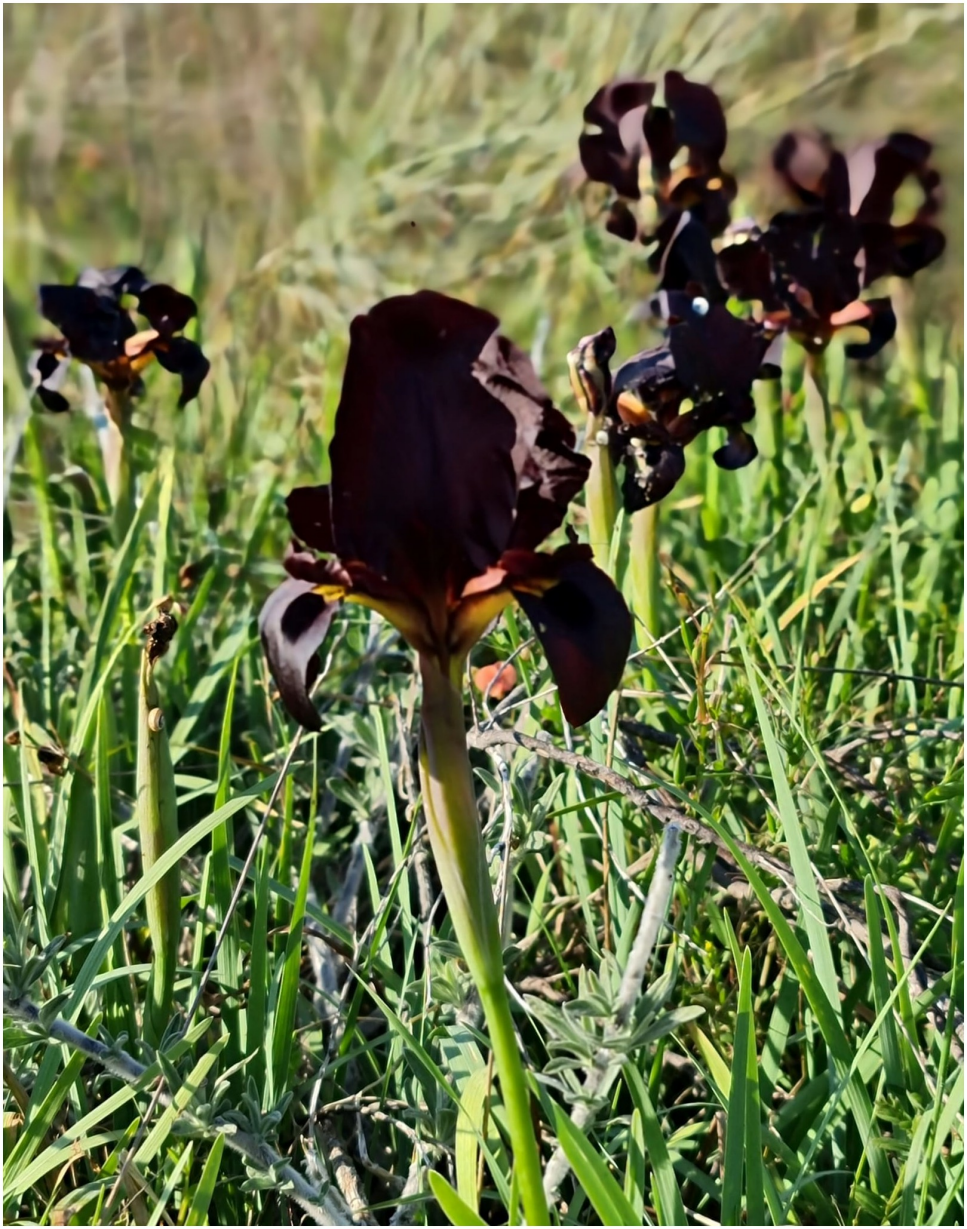
Black Iris, Ness Ziona; photograph: Oren Perez.

Fig 5 shows the distribution of visitors for each day (Fig 5(a)) and the average utility (Fig 5(b)) for the full-information scenario with learning after 100 seasons (N = 2000, *L* = 0.5). As can be seen, equilibrium is almost reached, and visitors enjoy positive utility almost every day (we ran a simulation with 1000 seasons and varying values of L, demonstrating similar results across different L values; see Figs 1, 2 and 6, technical supplement). Fig 5(c) reports the results of an analysis of the preferences of players who visited each day. It demonstrates that in an equilibrium state, the average utility of players who visited is higher than that of the players who stayed at home. The equilibrium, however, is not a social optimum because some players with a preference as low as 1.37 visited the reservation while others with a preference as high as 2.98 stayed at home (see p. 3 in the supplementary materials for complete analysis).

**Fig 7:**
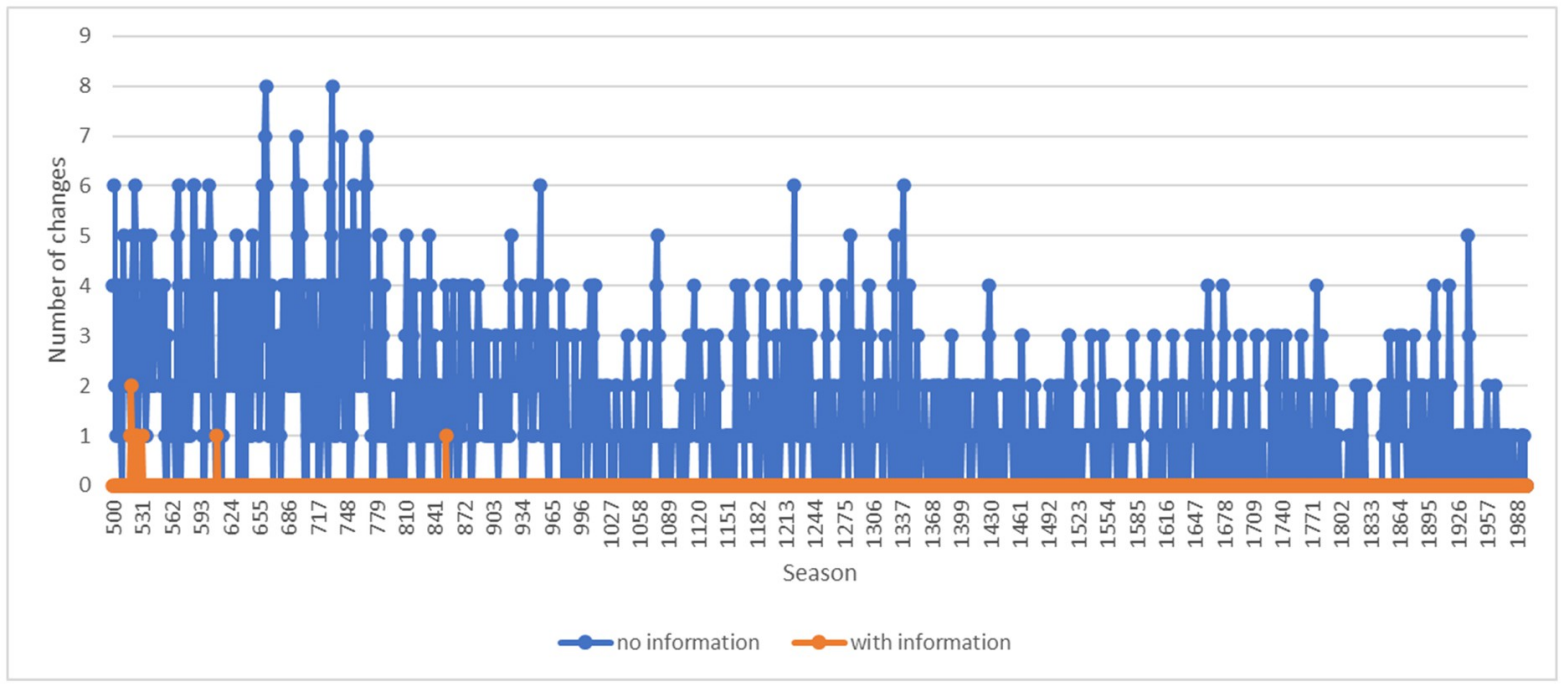
Stability and behavioural change in the no and with information conditions (with learning).

An important distinction between the no-information and full-information conditions with learning, is that adding learning to the full-information condition leads to a stable situation where some fraction of the population visits the reserve while the rest never do, whereas in the no-information condition with learning, some players continue to change their choices, but the probability of change becomes lower and lower and converges to zero. This is shown in Fig 7.

**Fig 8:**
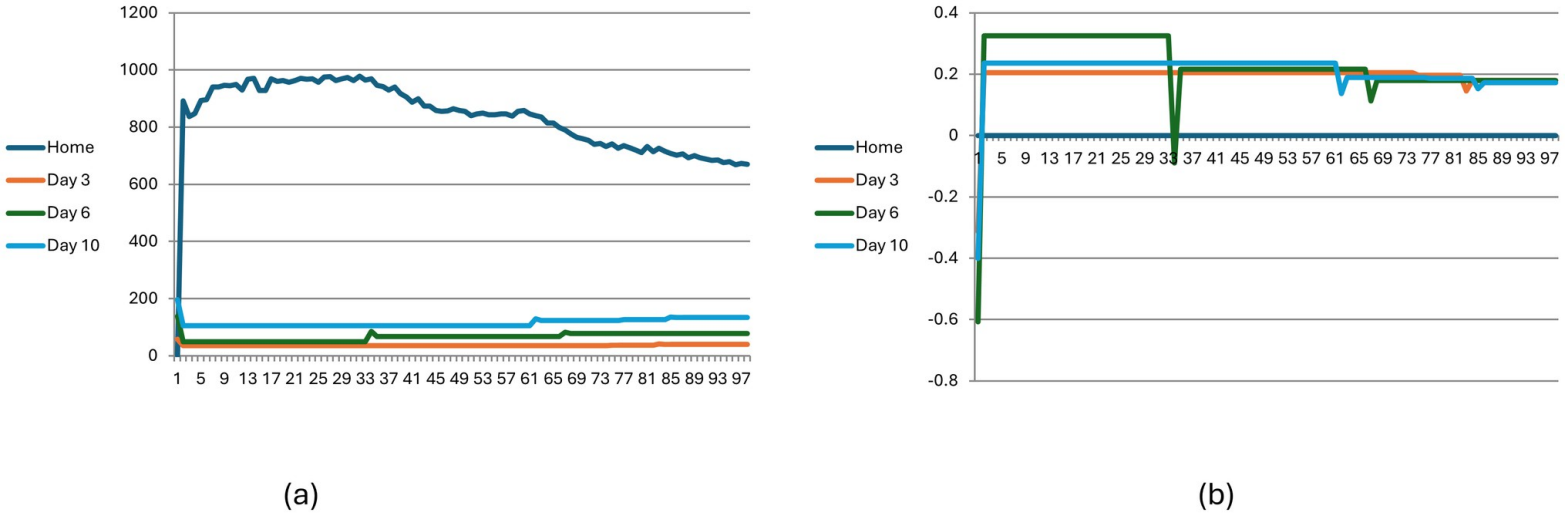
Visitation dynamic in the full-information with learning condition across 100 seasons (N = 2000, L = 0.5). Fig 8(a) describes the number of visitors on selected days; Fig 8(b) describes average utility.

Fig 8 describes the number of visitors (Fig 8(a)) and the average utility (Fig 8(b)) for visitors on selected days for the information model with learning during the first 100 seasons (N = 2000, *L* = 0.5).

We also tested another scenario (in the with information with learning condition) in which new agents, with no memory of the game’s history, replace part of the population (simulating potential migration or turnover). We tested two scenarios with 10 and 20 percent change. We found that the introduction of new agents prevents the game from converging. However, average utility remains positive for most players in the 10 percent scenario, but the percentage of players with positive utility decreases significantly as the number of new entrants increases. We provide detailed graphs of this analysis in the technical supplement.

### Testing the model against real data: the behaviour of bats around waterholes

To validate our model, we searched the literature for data on which our model can be tested. We found a useful testbed in a study conducted by Adams and Thibault on the behaviour of bat populations in a water-limited environment [25]. They conducted a fine-grain analysis of overlapping visitation times at waterholes to test the hypothesis that bat species temporally partition the use of water holes that are too small to accommodate spatial separation and simultaneous use. Their findings indicate that ‘bats drinking at small waterholes structure use of the resource on several levels, one of which is to temporally space visitations across species, perhaps to avoid overcrowding’ [25: 470]. Their research constitutes an empirically verified example of mammals using fine-grain temporal partitioning to facilitate coexistence in water-stressed environments. We used the field setting of their study as the empirical basis of the simulation. The research considered a single waterhole that was shared by five myotis species, a closely related group of ecologically similar species. The study focused on a period of 5.5 hours that started 30 minutes before sunset [25: 467]. Data on arrival times were categorized into 10-minute intervals (time was converted to minutes after sunset (MAS)).

We used our model of no-information with learning (with some necessary modifications) to test whether it can yield an equilibrium like the one demonstrated by Adams and Thibault. The primary modification we introduced was that each bat type has a slight preference to be with its own type. The game unfolds according to the following rules.

After the first round, each bat, *i*, collects a utility, calculated using the formula:

$$R=f\left(d_{i},n\left(d_{i}\right),C_{i},\;q_{type},\;z\right)$$ (6)

where *d*_*i*_ is the time slot (MAS) chosen by bat *i* and *n*(*d*_*i*_) is the number of bats that chose the same time slot (i.e., *d*_*i*_). As in the original model, *f* decreases as n(d_i_) increases and becomes negative for a large *n*(*d*_*i*_), depending on an exogenous variable, *z*, which represents the maximum number of bats that can jointly access the waterhole. We also assume that the utility of the bat is proportional to the percentage of bats of similar type that visit the waterhole in the same time slot. We assume that if the number of bats visiting the waterhole surpasses threshold *z*, which is identical for each bat, the utility of that bat becomes negative: *f* = −*C*_*i*_. *C*_*i*_ represents the energy wasted by the bat if it arrives at the waterhole but cannot access the water. The following rules describe the utility that bats receive as a function of the number of other attending bats. We assume that the amount of water remains constant throughout the day and is refilled between days.


$$q_{type}=n\%\;for\;each\;i$$ (7)

$$\begin{array}{l} f\left(d_{i}\neq 0,n\left(d_{i}\right),q_{type},C_{i},\;z\right)=\frac{z}{n\left(d_{i}\right)}\cdot \frac{n_{type\left(i\right)}}{n\left(d_{i}\right)}\;\text{if}\;\frac{z}{n\left(d_{i}\right)}\cdot \frac{n_{type\left(i\right)}}{n\left(d_{i}\right)}\geq 1.\;\text{Otherwise}\\ f\left(d_{i},n\left(d_{i}\right),C_{i},\;z\right)=-C_{i}\;(\text{that}\;\text{is},\;\text{if}\;\text{the}\;\text{number}\;\text{of}\;\text{bats}\;\text{arriving}\;\text{is}\;\text{larger}\;\text{than}\;\text{the}\\ \text{capacity}\;\text{of}\;\text{the}\;\text{waterhole}) \end{array}$$ (8)

$$f\left(d_{i}=0,n\left(d_{i}\right),C_{i},\;z\right)=0\;(\text{if}\;\text{the}\;\text{bat}\;\text{remains}\;\text{at}\;\text{its}\;\text{roost}\;\text{it}\;\text{receives}\;\text{no}\;\text{utility})$$ (9)

We assume that the bats have access only to data regarding the number of bats who were present at their chosen MAS. We further assume that the bats use the following strategy: each bat starts by randomly choosing a time slot after sunset (an MAS). Each MAS is assigned an equal probability. After each day, the bat assesses its utility. If the utility is positive, it continues with the *same choice* the next day. If the utility is zero or negative, it chooses its behaviour for next day based on the following rule:

it chooses not to visit the waterhole with probability 1 − *p*, and with probability *p* chooses a random MAS that does not include staying at the roost.

We incorporated the same learning mechanism we used in the previous simulations: if the bat experienced negative utility, it multiplies *p* by a constant *L<1* (set in the simulation to 0.5). The learning mechanism means that bats become increasingly less inclined to explore other visiting days after repeatedly experiencing negative results.

**Fig 9:**
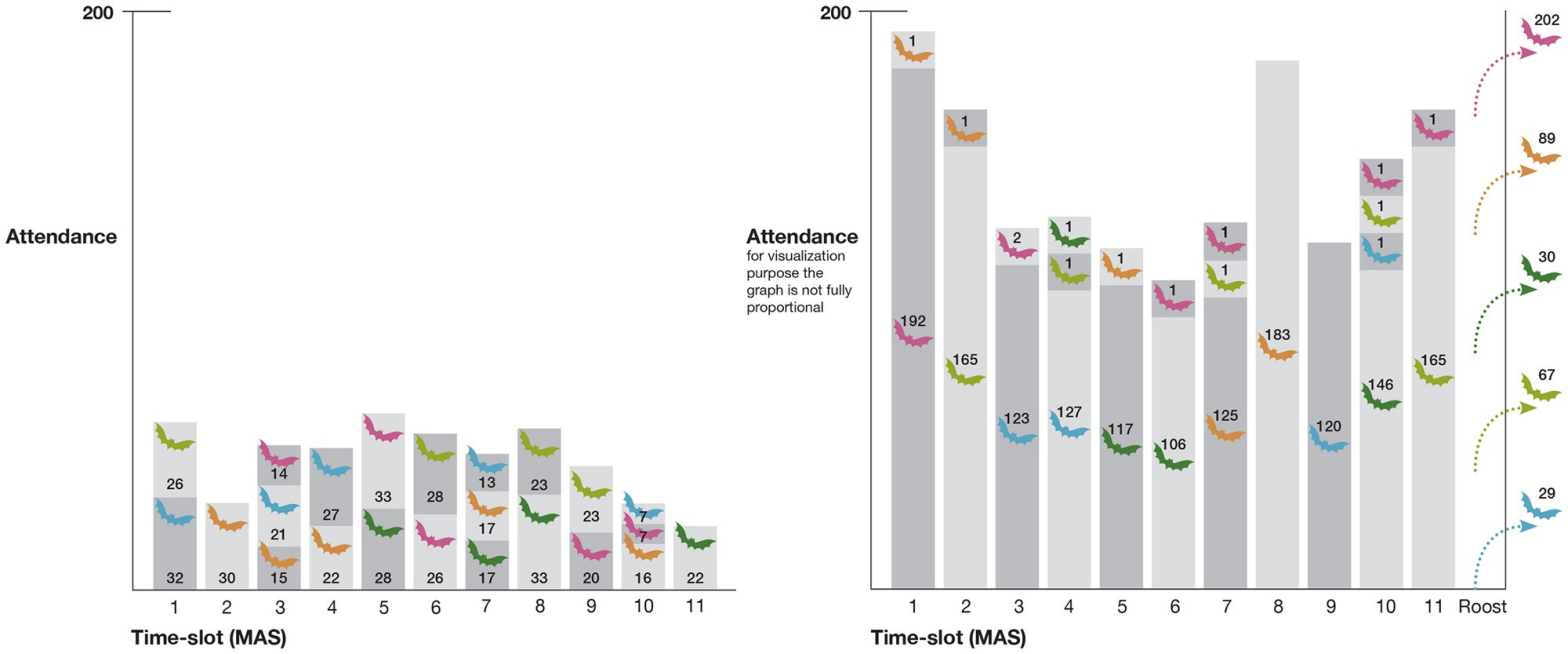
Bat’s temporal visitation distribution per type. Fig 9(a) and Fig 9(b) show the results of the simulation for 500 and 2,000 bats, respectively, with 11 time slots after 100 days.

Fig 9(a) and 9(b) show the results for 500 and 2,000 bats, respectively, with 11 time slots after 100 days. In both cases, the bats manage to partition themselves into the different time slots, where they also self-separate by types, almost all time slots having one or at most two different types of bats. In the 2,000 case, with the increasing congestion pressure due to the higher demand that exceeds the capacity of the waterhole, each time slot is dominated by a single type of bat. Moreover, because of the heightened congestion pressure in the 2,000 case, some bats are pushed out and need to look for alternative water sources (see p. 7 in the supplementary materials for the raw data for both figures).

## Discussion

To situate our work within the broader landscape of agent-based modeling (‘**ABM’**), it is instructive to consider the framework proposed by Riccardo Boero and Flaminio Squazzoni [26]. They distinguish between three types of agent-based models: ‘case-based models,’ which target specific empirical phenomena with a defined space-time context; ‘typifications,’ which target a specific class of empirical phenomena sharing some idealized properties; and ‘theoretical abstractions,’ which target a wide range of general phenomena without direct reference to reality. Our model falls within the category of ‘typifications,’ as it aims to develop better understanding of the dynamics of a general class of social dilemmas: temporal partitioning congestion games. The specific case used in our model—the Black Iris Game—serves as a heuristic anchor to illustrate the general dynamics of TPGs as a distinctive class. Our aim is not to make predictions about the specificities of the Black Iris game, but rather to explore the general principles governing TPGs. Our model shares similarities with the ’theoretical abstractions’ category, as we use it to examine, in an abstract theoretical manner, the distinction between complexity-based and game-theoretic analytic modeling approaches, as well as to critique the level-k approach to the depth-of-reasoning problem.

We begin with a general summary of our results, then discuss their theoretical implications and policy ramifications, before concluding with limitations and future research directions. Generally, we found that for both the no-information and with-information conditions, when there is no learning, and assuming the number of players is larger than the capacity of the iris habitat, the game does not converge, and an oscillatory dynamic develops (if the number of players is small enough so that they can all visit the reserve within the capacity limits of a single season, equilibrium is reached provided that the players use randomization in their strategies, as we assume here, even without learning). Adding information does not resolve the diabolic nature of this game. However, incorporating learning into both the no-information and with-information conditions leads to convergence. An important distinction between these conditions is that adding learning to the with-information condition results in a stable equilibrium where a fraction of the population visits the reserve while the rest never do. In contrast, in the no-information condition with learning, some players continue to change their choices, but the probability of change decreases over time and eventually converges to zero. The provision of information leads therefore to a more robust equilibrium but at the cost of causing social separation in the use of the common pool. Although the no-learning models fail to achieve equilibrium, they offer one advantage: a fairer utility distribution. Players may visit the flowers and experience positive utility for several seasons until an overflow of visitors occurs, causing some players to forgo the next season, allowing new players to visit (this outcome, however, depends on the size of *p*: if *p* is too large, it could increase the frequency of oscillations, causing a loss of utility for all). We also tested another scenario (in the with information with learning condition) in which new agents, with no memory of the game’s history, replace part of the population. We found that the introduction of new agents prevents the game from converging. This outcome can be averted by assuming that new entrants acquire knowledge of the game’s history through a socialization process within their community. Our model and results also have broader theoretical implications. First, we demonstrated the advantages of the computational approach compared to the game-theoretical one. A major limitation of the analytical approach is its inability to offer insights into the social dynamics that determine whether the system will converge to one of the multiple equilibria identified through analysis. In contrast, the computational approach enabled us to illuminate the dynamics of TPGs, thus surpassing the constraints of the analytical method.

Second, we would like to distinguish our approach to modeling the destructive meta-reasoning feature of TPGs from the level-k reasoning model, and shed light on its limitations. A standard level-*k* model assumes that the population is partitioned into types that differ in their *depth of reasoning* [27]. A level-0 type is nonstrategic and follows a simple decision rule that can be based on a uniform distribution over the set of strategies or some salient aspect of the game (e.g., the existence of a peak blossom day, in our case). A level-k type (*L*_*k*_), behaves as if the player provides the best response to the belief that the other player is a level *k-1* type. Thus, given a particular game, the model is characterized by: (a) an *L*_*0*_ behaviour, which is the starting point for iterative reasoning, and (b) a distribution of types.

Theoretically, this model can capture the reflexive structure of the Black Iris game and similar TPGs, where players reflect on each other’s thoughts. However, employing this framework to capture the dynamics of the Black Iris game does not seem to generate fruitful insights. Let us assume that the blossom season lasts for 2x+1 days. The peak days occur at day *x* + 1, and the community consists of *2x+1* types of equal distributions *L*_*0*_, *L*_*1*_, *L*_*2*_*… L*_*(2x-1)*_. In this scenario, L_0_ picks the focal day of maximal blossom (x+1). *L1*, responds to the choice by randomly choosing between x and x+2, and so on. This could lead to NE, with each type populating a day if the number of players of each type perfectly fits the maximum capacity of each day. In reality, it is unlikely that the distribution of K types would optimally fit the capacity of the different time slots, generating a destructive oscillatory behaviour and leading to poor results for all players. Previous research has found that the most frequent types are *L*_*0*_, *L*_*1*_, and *L*_*2*_ [28]. People find it extremely difficult to go deeper than three levels in their reflexive reasoning. If we assume the existence of three types, this could generate indefinite oscillation in situations where the time partition is larger than three. Overall, we do not think that the K-level model provides a useful framework for simulating the dynamic of such games. This conclusion reflects a more general problem: deterministic strategies taken by each player are likely to lead to poor general results because of reduced utility resulting from many players making the same decision. This is a well-known result in game theory and distributed computing, and it is usually solved by introducing randomness into the players’ reasoning [29].

Based on the above discussion, it seems that the most likely approach of non-naive players to the strategic complexity of TPGs, especially under conditions of over-crowdedness, is to incorporate some measure of randomness into their strategy. This reflects the fact that in the absence of external coordination, there is simply no way in which you can second-guess all the other players. Any attempt of a player to out-predict the others is likely to lead to infinite regress: a descent into a mental *rabbit hole* [14]. Thus, our model assumes that all the players have an equal level of reasoning depth (which we leave unspecified) that is reflected by using randomness in the strategies.

How would different types of regulatory intervention affect the players’ behaviour? Introducing fees will change the utility function without affecting the other parameters of the problem. Adding fees will shift the utility function and change the resulting equilibrium quantitatively but not qualitatively. The outcome, either way, will be fewer visitors to the reserve, with no improvement in coordination. This result may be problematic from a distributive justice perspective by permanently preventing people who could have enjoyed the reserve from visiting. Using a lottery based on pre-registration achieves the benefits of equilibrium, including the benefit of visitor diversity, without the need to share information. But this requires stronger involvement of an external regulator. It could also lead to an inferior equilibrium that is far from the social optimum because people with high preferences might lose the lottery and people with low preferences might be exposed to excessively high congestion levels.

Finally, we would like to address several limitations of our analysis that reflect its relatively abstract nature. While we grounded our model in several empirical cases from different contexts (natural recreation goods, publication slots in academic journals, and waterholes in arid regions), we have not attempted to validate it comprehensively across all these domains using actual data. We provided a partial validation of our model using data from research on bats’ behavior in a water-limited environment. However, this validation had a limited goal: we aimed to demonstrate the model’s general plausibility (its capacity to generate results like those observed in the field). We did not attempt to calibrate the model to provide accurate predictions in any of the domains on which it was grounded. While our primary goal is theoretical—to gain a better understanding of the dynamics of temporal partitioning games as a distinct class—it could still be beneficial to obtain data on human behavior in TPGs. Such data could allow us to corroborate and revise our modeling of players’ strategies, particularly in terms of how we model learning. In a recent paper Wijermans *et al* proposed to combine ABM with controlled behavioural experiments [30]. We believe this approach offers a promising path. Conducting behavioral experiments in a TPG, for example, could be instructive in understanding the strategies human participants use in such contexts. This empirically grounded understanding could then be applied to revise the TPG simulation.

## Supporting information

S1 File Supplementary analysis, S1-S5 Figs, S1-S3 Tables. (DOCX)

S1 Code Code for the general model. (TXT)

S2 Code Code for the general model. (TXT)
