# Supplementary material for: Emergent coordination in temporal partitioning congestion games
Source: PLoS One. 2024 Aug 19;19(8):e0308341. doi: 10.1371/journal.pone.0308341 (PMC11332916; doi:10.1371/journal.pone.0308341)
Supplement: S1 File — (DOCX) [file pone.0308341.s001.docx]

**Supplementary materials for**

# **Emergent Coordination in Temporal Partitioning Congestion Games**

**Reuven Cohen^1^ and Oren Perez^2^**

**19 March 2024**

**This file includes:**

Supplementary Text

Figs. S1 to S5

Tables S1 to S3

**Table S1 describes how the population distribution changes in the *No-information condition with learning between* seasons 900 and 1000.**

| Day | Season 900 | Season 1000 |
| --- | --- | --- |
| Home | 513 | 510 |
| 1 | 13 | 14 |
| 2 | 29 | 29 |
| 3 | 44 | 44 |
| 4 | 59 | 59 |
| 5 | 75 | 75 |
| 6 | 90 | 90 |
| 7 | 104 | 105 |
| 8 | 120 | 120 |
| 9 | 135 | 135 |
| 10 | 151 | 151 |
| 11 | 136 | 136 |
| 12 | 120 | 120 |
| 13 | 104 | 104 |
| 14 | 88 | 88 |
| 15 | 75 | 75 |
| 16 | 58 | 59 |
| 17 | 44 | 44 |
| 18 | 28 | 28 |
| 19 | 14 | 14 |

Table 1. Number of visitors each day after 900 and 1000 seasons for the no-information scenario with learning (L=0.5).

Table S2 shows that the behavior depends very weakly on the value of L. Thus, if learning exists (L<1), the population settles into an equilibrium relatively fast.

**Table S2: 1000 seasons with information and learning history and final stat. (maybe put different values of L here)**

| **Day** | **number of visitors** | **mean utility** | **mean preference** | **min preference** | **max preference** |
| --- | --- | --- | --- | --- | --- |
| home | 602 | 0 | 1.3837 | 1.00049 | 2.9837 |
| 1 | 14 | 0.137202 | 1.92082 | 1.65631 | 2.19336 |
| 2 | 30 | 0.153245 | 2.29868 | 1.62775 | 2.95905 |
| 3 | 44 | 0.160677 | 2.35659 | 1.54803 | 2.99451 |
| 4 | 55 | 0.167513 | 2.3033 | 1.68713 | 2.93158 |
| 5 | 71 | 0.166575 | 2.36536 | 1.50452 | 2.9931 |
| 6 | 84 | 0.167055 | 2.33876 | 1.43524 | 2.99976 |
| 7 | 98 | 0.161236 | 2.25731 | 1.47992 | 2.99139 |
| 8 | 111 | 0.141499 | 1.9633 | 1.4566 | 2.9566 |
| 9 | 125 | 0.165724 | 2.30173 | 1.48547 | 2.99695 |
| 10 | 138 | 0.165298 | 2.28111 | 1.40051 | 2.99762 |
| 11 | 125 | 0.154091 | 2.38433 | 1.37091 | 2.99927 |
| 12 | 114 | 0.159837 | 2.27768 | 1.44464 | 2.99805 |
| 13 | 99 | 0.163017 | 2.30553 | 1.4455 | 2.99744 |
| 14 | 83 | 0.166453 | 2.3026 | 1.4881 | 2.96094 |
| 15 | 69 | 0.167223 | 2.30767 | 1.38782 | 2.98352 |
| 16 | 57 | 0.16083 | 2.29183 | 1.47998 | 2.98022 |
| 17 | 41 | 0.171522 | 2.34414 | 1.70203 | 2.95959 |
| 18 | 27 | 0.170592 | 2.30299 | 1.74738 | 2.99677 |
| 19 | 13 | 0.198569 | 2.5814 | 1.40991 | 2.98358 |

**Fig. S1: Results for full information and learning with 2000 players, L=0.5.**

**Fig. S2: Results for full information and learning with 2000 players, L=0.9.**

**Fig. S3: Results for full information and learning with 2000 players, L=0.1.**

**Table S3(a) Bats’ temporal visitation distribution by type (n=500)**

| Time slot | Total | Type 1 | Type 2 | Type 3 | Type 4 | Type 5 | Average utility |
| --- | --- | --- | --- | --- | --- | --- | --- |
| 0 | 0 | 0 | 0 | 0 | 0 | 0 | 0 |
| 1 | 58 | 0 | 0 | 32 | 0 | 26 | 0.174259 |
| 2 | 30 | 30 | 0 | 0 | 0 | 0 | 0.666667 |
| 3 | 50 | 15 | 0 | 21 | 14 | 0 | 0.13792 |
| 4 | 49 | 22 | 0 | 27 | 0 | 0 | 0.206207 |
| 5 | 61 | 0 | 28 | 0 | 33 | 0 | 0.165036 |
| 6 | 54 | 0 | 0 | 0 | 26 | 28 | 0.185439 |
| 7 | 47 | 17 | 17 | 13 | 0 | 0 | 0.143899 |
| 8 | 56 | 0 | 33 | 0 | 0 | 23 | 0.184266 |
| 9 | 43 | 0 | 0 | 0 | 20 | 23 | 0.23369 |
| 10 | 30 | 16 | 0 | 7 | 7 | 0 | 0.262222 |
| 11 | 22 | 0 | 22 | 0 | 0 | 0 | 0.909091 |

**Table S3(b) Bats’ temporal visitation distribution by type (n=2000)**

| Time slot | Total | Type 1 | Type 2 | Type 3 | Type 4 | Type 5 | Average utility |
| --- | --- | --- | --- | --- | --- | --- | --- |
| 0 | 417 | 29 | 67 | 30 | 89 | 202 | 0 |
| 1 | 193 | 0 | 0 | 0 | 1 | 192 | 0.097375 |
| 2 | 166 | 0 | 165 | 0 | 1 | 0 | 0.113011 |
| 3 | 125 | 123 | 0 | 0 | 0 | 2 | 0.138921 |
| 4 | 129 | 127 | 1 | 1 | 0 | 0 | 0.134765 |
| 5 | 118 | 0 | 0 | 117 | 1 | 0 | 0.158156 |
| 6 | 107 | 0 | 0 | 106 | 0 | 1 | 0.174093 |
| 7 | 127 | 0 | 1 | 0 | 125 | 1 | 0.136811 |
| 8 | 183 | 0 | 0 | 0 | 183 | 0 | 0.10929 |
| 9 | 120 | 120 | 0 | 0 | 0 | 0 | 0.166667 |
| 10 | 149 | 1 | 1 | 146 | 0 | 1 | 0.108743 |
| 11 | 166 | 0 | 165 | 0 | 0 | 1 | 0.113011 |

**Fig. S4:** **Attendance (over 1000 seasons) and mean utility (last 50 seasons of the 1000 season simulation), full information and learning (**$\boldsymbol{L=0.5}$**) with 10% population turnover per season**

Attendance (over 1000 seasons) and mean utility (last 50 seasons of the 1000 season simulation) for the model with a population of 2000, full information and learning ($L=0.5$) with 10% population turnover per season. It can be seen that equilibrium (even weak equilibrium) is never reached. However, except for the visitors on the first and last day, most visitors experience positive utility.

**Fig. S5:** **Attendance (over 1000 seasons) and mean utility (last 50 seasons of the 1000 season simulation), full information and learning (**$\boldsymbol{L=0.5}$**) with 20% population turnover per season**

Attendance (over 1000 seasons) and mean utility (last 50 seasons of the 1000 season simulation) for the model with a population of 2000, full information and learning ($L=0.5$) with 20% population turnover per season. It can be seen that equilibrium (even weak equilibrium) is never reached. Compared to the 10% turnover case, many more visitors experience negative utility.
